# Combination Therapy with Local Radiofrequency Ablation and Systemic Vaccine Enhances Antitumor Immunity and Mediates Local and Distal Tumor Regression

**DOI:** 10.1371/journal.pone.0070417

**Published:** 2013-07-24

**Authors:** Sofia R. Gameiro, Jack P. Higgins, Matthew R. Dreher, David L. Woods, Goutham Reddy, Bradford J. Wood, Chandan Guha, James W. Hodge

**Affiliations:** 1 Laboratory of Tumor Immunology and Biology, Center for Cancer Research, National Cancer Institute, National Institutes of Health, Bethesda, Maryland, United States of America; 2 Center for Interventional Oncology, Radiology, and Imaging Sciences, National Institutes of Health, Bethesda, Maryland, United States of America; 3 Department of Radiation Oncology, Montefiore Medical Center, Albert Einstein College of Medicine, New York, New York, United States of America; Federal University of São Paulo, Brazil

## Abstract

**Purpose:**

Radiofrequency ablation (RFA) is a minimally invasive energy delivery technique increasingly used for focal therapy to eradicate localized disease. RFA-induced tumor-cell necrosis generates an immunogenic source of tumor antigens known to induce antitumor immune responses. However, RFA-induced antitumor immunity is insufficient to control metastatic progression. We sought to characterize (a) the role of RFA dose on immunogenic modulation of tumor and generation of immune responses and (b) the potential synergy between vaccine immunotherapy and RFA aimed at local tumor control and decreased systemic progression.

**Experimental Design:**

Murine colon carcinoma cells expressing the tumor-associated (TAA) carcinoembryonic antigen (CEA) (MC38-CEA^+^) were studied to examine the effect of sublethal hyperthermia *in vitro* on the cells’ phenotype and sensitivity to CTL-mediated killing. The effect of RFA dose was investigated *in vivo* impacting (a) the phenotype and growth of MC38-CEA^+^ tumors and (b) the induction of tumor-specific immune responses. Finally, the molecular signature was evaluated as well as the potential synergy between RFA and poxviral vaccines expressing CEA and a TRIad of COstimulatory Molecules (CEA/TRICOM).

**Results:**

*In vitro*, sublethal hyperthermia of MC38-CEA^+^ cells (a) increased cell-surface expression of CEA, Fas, and MHC class I molecules and (b) rendered tumor cells more susceptible to CTL-mediated lysis. *In vivo*, RFA induced (a) immunogenic modulation on the surface of tumor cells and (b) increased T-cell responses to CEA and additional TAAs. Combination therapy with RFA and vaccine in CEA-transgenic mice induced a synergistic increase in CD4^+^ T-cell immune responses to CEA and eradicated both primary CEA^+^ and distal CEA^–^ s.c. tumors. Sequential administration of low-dose and high-dose RFA with vaccine decreased tumor recurrence compared to RFA alone. These studies suggest a potential clinical benefit in combining RFA with vaccine in cancer patients, and augment support for this novel translational paradigm.

## Introduction

Thermal ablation techniques such as microwave, cryotherapy, laser ablation, high-intensity focused ultrasound (HIFU), and radiofrequency ablation (RFA) have been increasingly used for minimally invasive treatment of local unresectable tumors [1]. RFA has proven to be safe and certain systems are cleared by the U.S. Food and Drug Administration (FDA) for ablation of soft tissue, unresectable liver tumors, and painful lytic bone metastases. RFA has been successfully used to manage kidney, liver, breast, bone, and lung malignancies [1], [2], [3].

RFA employs an alternating RF current to generate frictional heat and induce coagulative necrosis within and surrounding solid tumors. This form of cell death is accompanied by release of inflammatory cytokines, and “danger signals” that can trigger and/or amplify pre-existing antitumor immunity [4], [5], [6]. Preclinical and clinical data suggest that RFA-induced necrosis is a source of tumor antigens that can be used by the host’s immune system to generate antitumor immunity [7]. However, immune responses elicited by RFA alone are insufficient for morphologically visible responses or systemic control of tumor [8], [9].

Vaccine immunotherapy is an approach under active investigation in both preclinical and clinical settings for a wide spectrum of human cancers. Accumulating clinical evidence indicates that vaccine immunotherapy is safe and may have the greatest clinical benefit when targeted against residual disease [10]. The FDA recently approved the first therapeutic vaccine for prostate cancer (PROVENGE®; Dendreon, Seattle, WA). In this study, active vaccination was combined with RFA, to attempt to augment the immune response of each treatment. RFA was combined with active immunotherapy with tumor-associated antigen (TAA)-specific poxviral vaccine regimens to induce and potentiate T-cell responses against carcinoembryonic antigen (CEA). We hypothesized that the dose of RFA energy could be tailored to induce immunogenic modulation of the tumor or to promote an immunogenic microenvironment, to make tumor more prone to T cell-mediated killing, and transform it into an effective *in situ* antigen sink able to synergize with vaccine to promote effective tumor control and reduce recurrence. To test this hypothesis, we examined (a) the effect of mild hyperthermia on tumor phenotype and sensitivity to T cell-mediated lysis, (b) the effect of RFA dose on tumor burden, phenotype, and generation of immune responses to non-vaccine encoded (cascade) antigens, (c) the molecular signature induced by RFA, and (d) the potential synergy between RFA and vaccine to elicit antitumor immune responses able to promote effective tumor control of both primary and distant antigen-disparate metastases. We further sought to exploit the immune adjuvant potential of sequential delivery of low-dose (sub lethal) and high-dose (lethal) RFA to synergize with vaccine to promote effective antitumor immunity and increase durable complete responses (CRs). In clinical practice, a form of low dose RFA occurs during the ramp up to high dose RFA, and also occurs at the periphery of the ablative high dose RFA, due to spatial attenuation of thermal conduction at the periphery of a thermal lesion [11].

These findings support the therapeutic potential of combining RFA with vaccine therapy to promote both local and systemic anti-tumor effects.

## Materials and Methods

### Recombinant Poxviruses

The rMVA, rV and rF viruses containing the human CEA gene under control of the 40 k promoter and the murine B7.1, ICAM-1, and LFA-3 genes (designated rMVA, rV-CEA/TRICOM and rF-CEA/TRICOM, respectively) have been previously described [12], [13]. The rF virus containing the gene for murine granulocyte-macrophage colony-stimulating factor (GM-CSF) under control of the 40 k promoter has also been described [14].

### Ethics Statement

This study was carried out in strict accordance with the recommendations in the Guide for the Care and Use of Laboratory Animals of the National Institutes of Health. The protocol was approved by the National Cancer Institute Animal Use and Care Committee (ASP Number: LTIB-51). All efforts were made to minimize suffering. All experimental animals were monitored daily by trained animal caretakers. Animals that reached humane endpoints were euthanized by cervical dislocation. Cervical dislocation was be used to euthanize animals whose body weight reduce to 15% of normal, have difficulty breathing, or are cachectic. Mice were weighed twice a week. Any animal experiencing rapid weight loss, debilitating diarrhea, rough hair coat, hunched posture, labored breathing, lethargy, persistent recumbence, jaundice, anemia, significantly abnormal neurological signs, bleeding from any orifice, self-induced trauma, impaired mobility, becomes moribund or other wise becomes unable to obtain food or water was immediately euthanized.

### Animals and Cells

Female C57BL/6 mice were obtained from the National Cancer Institute, Frederick Cancer Research Facility (Frederick, MD). CEA-transgenic (CEA-Tg) C57BL/6 mice have been previously described [15]. These studies used murine colon adenocarcinoma cells expressing human CEA (MC38-CEA^+^) [16]. Before transplantation to mice, tumor cells were trypsinized, dispersed through a 70-µm cell strainer (Falcon; Becton Dickinson, Franklin Lakes, NJ), and washed twice in PBS before final suspension in PBS. Colon carcinoma SW620 cells were obtained from American Type Culture Collection (Manassas, VA) and cultured in media designated by the provider for propagation and maintenance. Cells were incubated at 37°C with 5% CO_2_.

### Peptides

The peptide CEA_526–533_ (EAQNTTYL), designated CEA_526_, is an H-2D^b^-binding CEA-specific CD8 epitope that has been previously characterized [17]. The H-2K^b^-binding peptide p15e_604–611_ (KSPWFTTL), designated here as p15e_604_, has been described [12]. The H-2D^b^-restricted peptide p53_232–240_ (KYMCNSSCM), designated here as p53_232_, and the H-2K^b^-restricted peptide survivin_57–64_ (QCFFCFKEL) have been previously described [18], [19]. The vesicular stomatitis virus peptide VSV-N_52–59_ (RGYVYQGL) was used as a control [20].

### CEA- and gp70-specific T cells

The H-2D^b^-restricted, CEA-specific CD8^+^-CTL line, designated CAP-M8, recognizes the peptide epitope CEA_526_ and has been previously described [17]. The H-2K^b^-restricted, gp70-specific CD8^+^-cytotoxic T cells, here designated gp70, recognizes the peptide p15e_604_ and has been previously described [12]. The HLA-A2-restricted, CEA-specific, CD8^+^ cytotoxic T-cell line, designated CEA CTL, recognizes the CEA peptide epitope YLSGANLNL (CAP-1) [21]. It was maintained and propagated as previously described [22].

### In Vitro Studies

#### Cellular growth

Adherent MC38-CEA^+^ cells in log-growth phase were exposed *in vitro* to 37°C or 42°C for 1 h in incubators with 5% CO_2_. Tumor cells were incubated at 37C/5%CO2 additional 4 days. Cells were harvested daily and viable cells were counted by trypan blue exclusion using a Cellometer Auto T4 automated cell counter (Nexcelom Bioscience, Lawrence, MA).

#### Phenotypic analysis

To investigate the effects of *in vitro* exposure of tumor cells to hyperthermia on the expression of immune-relevant proteins, adherent MC38-CEA^+^ murine tumor cells were harvested 24 and 48 h after exposure to 37°C or 42°C for 1 h in 5% CO_2_. Tumor cell surfaces were stained using the primary labeled monoclonal antibodies (mAbs) CD95(Fas)-PE, CD54(ICAM-1)-FITC, H-2K^b^-FITC, and H-2D^b^ FITC (BD Biosciences, San Diego, CA). The appropriate isotype-matched controls were purchased from BD Biosciences. The anti-CEA mAb COL-1-FITC has been described [23]. Proteins were scored as up-regulated if detection levels or mean fluorescence intensity (MFI) increased by ≥30% or ≥50% following hyperthermia treatment, respectively. Stained cells were acquired on a FACScan flow cytometer using CellQuest software (BD Biosciences). Isotype control staining was <5% for all samples analyzed. Cell viability was >94%. Dead cells were excluded from the analysis based on side scatter profile.

#### Cytotoxicity assays

To evaluate the effect of hyperthermia on the sensitivity of murine tumor cells to CTL-mediated killing, MC38-CEA^+^ cells were exposed *in vitro* to 37°C or 42°C for 1 h in 5% CO_2_. After 24 h, MC38-CEA^+^ target cells were harvested and used as targets in a standard cytotoxicity assay with ^111^In [24], [25]. Cells were incubated at 37°C/5% CO_2_ at an effector:target ratio of 80∶1, using CAP-M8 and gp70 as effector cells. To examine the effect of hyperthermia and gamma radiation on sensitivity of human colon carcinoma cells to CTL-mediated lysis, SW620 cells were exposed *in vitro* to 37°C or 42°C for 1 h, or to gamma radiation (0 or 10 Gy). After 48 h, cells were harvested and incubated with CEA-specific effector T cells at an effector:target ratio of 30∶1. Data were averaged and depicted as percentage of lysis ± S.E.M.

### *In Vivo* Studies

#### Radiofrequency ablation

After animals were anesthetized with ketamine/xylazine, RFA was performed using a similar technique as previously described, using an RFA Pain Management System (Baylis, Montreal, CA) equipped with a RFA 22-gauge needle with 4 mm active tip inserted in the center of the tumor [26]. The dose of RFA was varied by exposing tumors for different lengths of time to 60–70°C in the center of the tumor. Tumors were 50–200 mm^3^.

#### Effect of ultra low-dose RFA on tumor phenotype and CEA-specific immune responses

Female C57BL/6 mice (*n* = 7) were injected s.c. on day 0 with 3×10^5^ MC38-CEA^+^ tumor cells. Tumors were treated with low-dose RFA (7 s; 60–70°C) on day 15 and excised 2 days post-treatment. Phenotypic analysis was performed in single-cell suspensions from pooled tumors after blocking Fc receptors with anti-CD16/CD32 (2.4 G2) antibody and labeling with mAbs specific for CEA (COL-1) [27], Fas (CD95), ICAM-1 (CD54), H-2K^b^, and H-2D^b^. Cellular immunofluorescence was analyzed as before. To evaluate CEA-specific T-cell immune responses, proliferation of pooled splenic CD4^+^ T cells in response to protein antigens was assessed 29 days after tumor transplant, as previously described [28]. The mean cellular proliferation of negative control responses was subtracted from proliferation in response to CEA and beta-galactosidase protein antigens. The data were averaged and depicted as delta cpm ± S.E.M.

#### Effect of RFA dose on tumor viability, phenotype, and T-cell immune responses

Female CEA-Tg mice (*n* = 8–10/group) were injected s.c. on day 0 with MC38-CEA^+^ tumor cells. On day 15, tumors were exposed to sham or increasing doses (30, 60, or 90 s) of RFA. For histologic studies, tumors were harvested 3 h post-RFA exposure and frozen in a liquid nitrogen bath. Tumor viability was assessed in histologic sections by tetrazolium staining [29]. Tumor dimensions in individual animals were measured using digital calipers, and tumor volumes were calculated as *L* ^2^×*W*/2, where *L* and *W* denote tumor length and width, respectively. On day 17, excised CEA^+^ tumor cells were evaluated by flow cytometry for expression of HSP70, ICAM-1, and TRAIL receptor 2 (TRAIL-R2), as described above. On day 29, purified CD4^+^ splenic T cells were tested for reactivity to CEA protein (25 µg/mL) in an *in vitro* lymphoproliferation assay as before. To evaluate CD8^+^ T-cell responses specific for TAAs, pooled splenocytes were stimulated with 1 ug/mL CEA_526_, p15e_604_, p53_232_, or survivin_57_. After 6 days, bulk lymphocytes were recovered by centrifugation through a Ficoll-Hypaque gradient. T cells were restimulated with CEA_526_, p15e_604_, p53_232_, survivin_57_, or the control peptide VSV-N_52_ for 24 h in the presence of freshly irradiated naive splenocytes. Supernatant was collected and analyzed for murine IFN-g by cytometric bead array (BD Pharmingen™; BD Biosciences) according to the manufacturer’s instructions.

### RFA and Vaccine Combination Therapy Studies

#### Metasynchronous tumor studies

To determine whether RFA could mediate abscopal regression of antigen-disparate tumors after systemic vaccination, CEA-Tg mice (*n* = 5–7) received MC38-CEA^+^ cells (3×10^5^) on day 0 (right flank s.c.; primary tumor) and MC38-CEA^–^ cells (3×10^5^) on day 5 (left flank s.c.; distal tumor). Vaccinated animals received PBS or 1×10^8^ pfu of rV-CEA/TRICOM admixed with 1×10^7^ pfu rF-GM-CSF on day 4 and 1×10^8^ pfu rF-CEA/TRICOM admixed with 1×10^7^ pfu rF-GM-CSF on days 11 and 18, alone or in combination with high-dose RFA. Untreated animals were used as controls. On day 13, only the MC38-CEA^+^ tumor was treated with RFA (30–300 s; 60–70°C). Volumes of both primary and distal tumors, calculated as described above, were assessed every 2–3 days using digital calipers.

#### Immune responses

To assess tumor-specific immune responses after combination therapy, CEA-Tg mice (*n* = 5–7) were transplanted with MC38-CEA^+^ tumor cells as before and received sham or high-dose RFA (30–300 s; 60–70°C) on day 15. Vaccinated animals received rMVA-CEA/TRICOM (1×10^8^ pfu s.c.) on day 4 and rF-CEA/TRICOM (1×10^8^ pfu s.c.) on days 11 and 18, alone or in combination with high-dose RFA. On day 39, an *in vitro* lymphoproliferation assay, as before, tested purified CD4^+^ splenic T cells’ reactivity to CEA protein (50 ug/mL).

#### Combination therapy with vaccine and sequential dosing of RFA

CEA-Tg mice (*n* = 25–29) received MC38-CEA^+^ cells (3×10^5^ s.c.) on day 0. Tumors received sham or low-dose RFA (10–30 s; 60–70°C) on day 12, followed by curative-intent high-dose RFA (30–300 s, 60–70°C) on day 15. Vaccinated animals received rMVA-CEA/TRICOM (1×10^8^ pfu s.c.) on day 4 and rF-CEA/TRICOM (1×10^8^ pfu s.c.) on day 11 and every 7 days thereafter, in combination with sham or high-dose RFA. Tumor volumes were determined as before.

#### Molecular array studies

CEA-Tg mice (*n* = 3) were transplanted with MC38-CEA^+^ cells on day 0. Tumors were exposed to RFA sham or RFA (30 s; 60–70°C) on day 13. Vaccinated animals received rMVA-CEA/TRICOM on day 4 and rF-CEA/TRICOM on day 11, alone or in combination with RFA. On day 16, tumors were harvested and microRNA transcript analysis was performed by SABiosciences (Valencia, CA) using the murine cancer microRNA PCR array MAM-102A. The array analyzed transcript expression changes pre- and post-treatment with RFA alone or combined with vaccine. Post-treatment, transcripts were considered to be up-regulated or down-regulated if their normalized intensity ratio was ≥5 or ≥0.2 (5-fold cutoff), as previously described [24].

#### Statistical analysis

Significant differences between treatment groups were determined by 1-way ANOVA with Tukey’s comparison based on a confidence interval of 95% using Prism 4.0c software (GraphPad Software Inc., La Jolla, CA). Alternatively, statistical differences between 2 treatments were analyzed by unpaired Student’s *t* test with a 2-tailed distribution and reported as *P* values. Significance for the proportion of CRs was determined by contingency analysis at 95% confidence by Fisher’s exact test. Differences in the distribution of flow cytometry analysis data were considered to be significant if ≥30% (% positive) or ≥50% (MFI).

## Results

### *In Vitro* Exposure of Murine and Human Tumor Cells to Hyperthermia Modulates Phenotype and Increases Sensitivity to CTL-Mediated Killing

Certain treatment modalities have been shown to induce immunogenic modulation of tumor by altering the expression of specific immune-relevant molecules that can render the tumor more sensitive to immune-mediated attack [10]. These modalities include some chemotherapeutic regimens [25], [30] and certain forms of energy delivery such as radiation [10], [31] and HIFU [32], [33]. Delivering radiofrequency energy to tumor lesions generates heat, which exposes the lesions to a gradient of thermal stress. In this study, we first investigated whether exposing a murine colon carcinoma cell line expressing CEA to hyperthermia-induced thermal stress affected cellular survival and proliferation. These levels of sublethal hyperthermia mimic the milieu at the periphery of a RFA treatment zone [11]. MC38-CEA^+^ cells were exposed *in vitro* to 42°C for 1 h. Control cells were exposed to 37°C for 1 h. To ascertain if this dose of 42°C for 1 h hyperthermia was sublethal, the total number of viable MC38-CEA^+^ cells was determined daily for 4 days post-exposure by trypan blue exclusion. Results indicated that this dose of hyperthermia had a small albeit significant effect on cellular growth relative to control (Fig. S1).

Next examined was the effect of hyperthermia on cell-surface expression of CEA, Fas, ICAM-1, and MHC class I molecules, each of which has been implicated in enhancing antitumor T-cell responses through diverse mechanisms [10], [34], [35]. MC38-CEA^+^ cells were exposed to 37°C or 42°C, as described above, and harvested 24 and 48 h after exposure to thermal stress. Exposure of tumor cells to hyperthermia significantly increased the population of CEA^+^ cells and CEA expression level per cell at both time points (Table 1). Expression of Fas and MHC class I protein on the surface of tumor cells also increased after exposure to hyperthermia. No significant changes in ICAM-1 expression were observed (data not shown).

**Table 1:** Effect of hyperthermia on phenotype of murine colon carcinoma cells.

		Percent Positive (MFI)			
Time	Treatment	CEA	Fas	H-2 Kb	H-2 Db
24 h	37°C	20.4% (13)	47.9% (12)	66.7% (12)	18.8% (12)
24 h	42°C	***54.4% (31)***	49.0% (17)	62.5% ***(18)***	18.9% (16)
48 h	37°C	32.9% (14)	58.7% (12)	74.9% (14)	35.6% (14)
48 h	42°C	***54.5% (24)***	66.3% ***(18)***	90.9% ***(30)***	***65.2% (21)***

MC38-CEA^+^ cells were exposed *in vitro* to 37°C or 42°C for 1 h. At 24 or 48 h post-exposure, cells were analyzed by flow cytometry for surface expression of CEA, Fas, MHC class I H-2K^b^ and H-2D^b^. Values in italic bold denote increase in protein expression ≥30% (% positive) or ≥50% (mean MFI) relative to control cells (37°C). Data is representative of two independent experiments.

**Figure 1:**
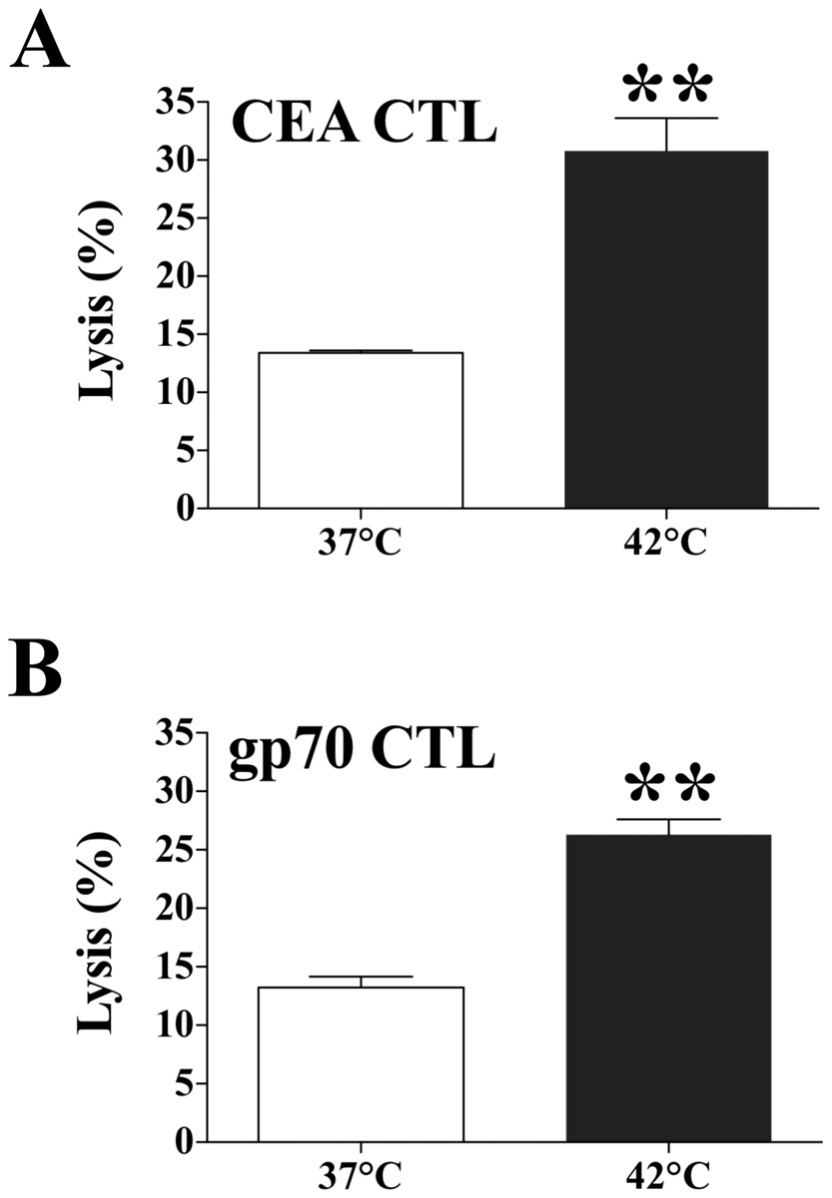
Effect of hyperthermia on sensitivity of murine colon carcinoma cells to CTL-mediated lysis. MC38-CEA^+^ cells were exposed *in vitro* to 37°C or 42°C for 1 h. Twenty-four hours after exposure, cells were harvested and labeled with ^111^In. The sensitivity of MC38-CEA^+^ target cells to **A.** CEA– or **B.** GP70-specific killing was determined after cells were incubated at an effector:target ratio of 80∶1. Results are presented as mean ± S.E.M. from 3 replicate wells. Asterisks denote statistical significance (*P*<0.004) relative to control cells (2-tailed *t* test). Data is representative of two independent experiments.

To evaluate the functional significance of phenotypic changes in tumor cells after exposure to hyperthermia, we examined the sensitivity of target MC38-CEA^+^ cells to CTL-mediated lysis after exposure to 37°C or 42°C. We used the CEA-specific CTL line CAP-M8, which recognizes the epitope CEA_526_ on carcinoma cells [17]. We also used a gp70-specific CTL line that recognizes the peptide p15e_604_ [12]. Murine MC38-CEA^+^ colon carcinoma cells positive for H-2D^b^, H-2K^b^, and CEA were exposed to 42°C or 37°C for 1 h. After 24 h a standard 18-h assay determined the sensitivity of MC38-CEA^+^ target cells to CAP-M8- and gp70-mediated killing. Exposing tumor cells to 42°C significantly enhanced both CEA_526_- and p15e_604_-specific CTL-mediated lysis relative to tumor cells exposed to 37°C (*P* = 0.004 and *P* = 0.001, respectively) (Fig. 1). These data indicate that exposure of murine tumor cells to sublethal doses of hyperthermia does not markedly affect either proliferation or cellular viability, but does up-regulate immune-relevant proteins and increase tumor sensitivity to CTL-mediated killing. This sublethal hyperthermia mimics the bioheat exchange process at the periphery of an RFA treatment zone [11].

**Figure 2:**
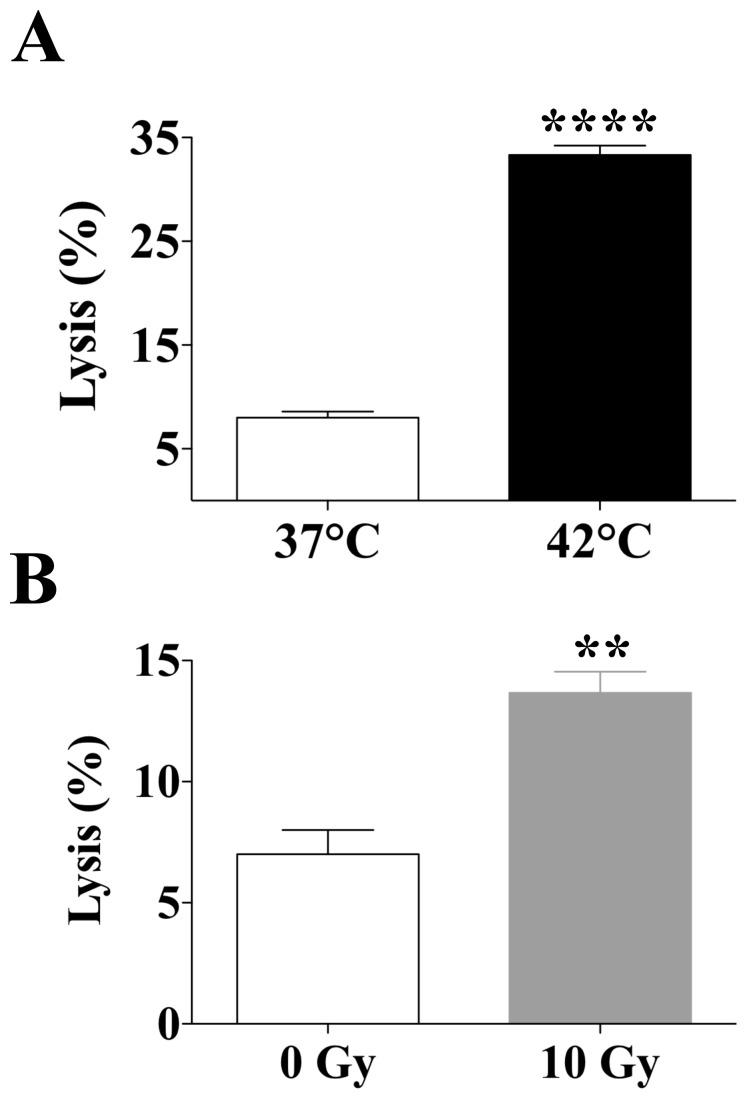
Effect of hyperthermia and gamma radiation on sensitivity of human colon carcinoma cells to CEA-specific CTL-mediated lysis. SW620 cells were exposed *in vitro* to **A**, 37°C or 42°C for 1 h, or to **B**, 0 or 10 Gy. Forty-eight hours post-exposure, cells were harvested and labeled with ^111^In. The sensitivity of SW620 target cells to CEA-specific killing was determined after cells were incubated with CEA-specific T cells at an effector:target ratio of 80∶1. Results are presented as mean ± S.E.M. from 3 replicate wells. Asterisks denote statistical significance (**P = 0.008; ****P<0.0001) relative to control cells (2-tailed *t* test). Radiation exposure data is representative of three independent experiments.

Next we sought to examine if these observations could be extended to human carcinoma cells and were comparable to the well-described enhancement in tumor sensitivity to CTL-mediated lysis mediated by another type of energy, gamma irradiation [24], [36]. SW620 cells were exposed to mild hyperthermia (37°C or 42°C), or to gamma radiation (0 or 10 Gy). After 48 h a standard 18-h assay determined the sensitivity of SW620 target cells to CEA-specific lysis by HLA-A2-restricted CD8^+^ cytotoxic T-cells that recognize the CEA peptide epitope CAP-1 [21]. Exposing tumor cells to 42°C significantly increased CEA-specific CTL-mediated killing relative to tumor cells exposed to 37°C (*P*<0.0001) (Fig. 2A). In addition, exposure of human colon carcinoma cells to radiation (10 Gy) significantly enhanced the sensitivity of SW620 cells to CEA-specific T-cell lysis (*P* = 0.008) (Fig. 2B). Together, these data indicate that exposure of human colon carcinoma cells to mild hyperthermia augments the sensitivity of human colon carcinoma cells to CTL-mediated killing and is comparable to that attained by gamma radiation.

### Ultra Low-Dose RFA Modulates Tumor Phenotype and CEA-Specific Immune Responses *In Vivo*

**Figure 3:**
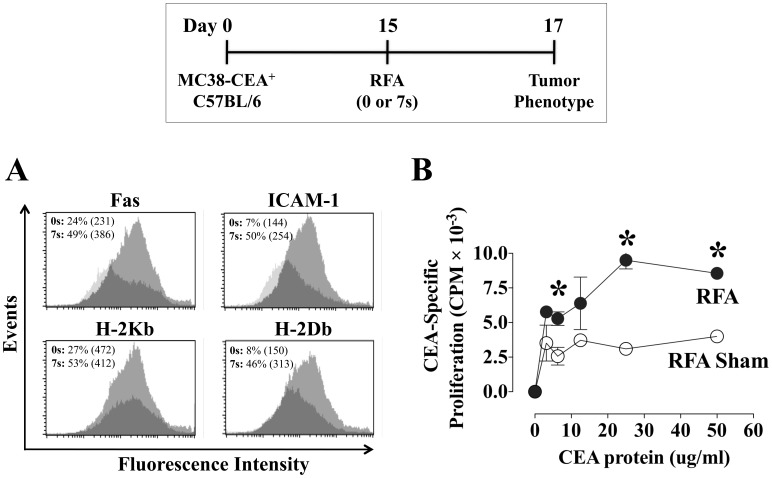
Tumor phenotype and CEA-specific immune responses after ultra low-dose RFA. Female C57BL/6 mice (*n* = 7/group) were injected s.c. on day 0 with MC38-CEA^+^ tumor cells. On day 15, tumors were exposed to RFA sham (0 s) or ultra low-dose RFA (7 s, 60–70°C). **A**, on day 17, the cell-surface phenotype of pooled excised tumor cells was analyzed by flow cytometry. Histograms depict fluorescence intensity of CEA^+^ tumor cells expressing Fas, ICAM-1, and MHC class I H-2K^b^ and H-2D^b^ before (dark histograms) and after (light histograms) RFA. Inset numbers represent % positive cells and MFI (parentheses) for each marker. **B**, on day 29, purified CD4^+^ splenic T cells from animals exposed to sham or low-dose RFA were tested by *in vitro* lymphoproliferation assay for reactivity to CEA protein (0–50 ug/mL). Results are depicted as mean CEA-specific CD4^+^ proliferation ± S.E.M. after subtraction of background CD4^+^ reactivity to control beta-galactosidase protein. Asterisks denote statistical significance between treatment groups (*P*<0.05, 2-tailed *t* test). Effect of RFA on tumor phenotype data was performed twice yielding similar results.

Next, we examined whether the phenotypic changes induced *in vitro* by exposure to sublethal hyperthermia could be captured by the gradient of thermal energy arising from treatment of MC38-CEA^+^ tumors with ultra-low dose RFA *in vivo*. On day 0, female C57BL/6 mice were transplanted with MC38-CEA^+^ tumor cells, and on day 15 tumors received sham or ultra low-dose RFA (7 s, 60–70°C). On day 17, the phenotype of pooled excised CEA^+^ tumor cells was analyzed by flow cytometry for cell-surface expression of Fas, ICAM-1, H-2K^b^, and H-2D^b^. As shown in Fig. 3A, ultra low-dose RFA exposure induced significant up-regulation of Fas, ICAM-1, and both MHC class I molecules in MC38-CEA^+^ tumors. Next we sought to examine if tumor exposure to ultra low-dose RFA could induce an anti-tumor immune response. On day 29, purified CD4^+^ splenic T cells from animals exposed to sham or ultra low-dose RFA were tested by *in vitro* lymphoproliferation assay for reactivity to CEA protein. As shown in Fig. 3B, control animals exposed to RFA sham had minimal CD4^+^-specific proliferation to CEA. However, tumor exposure to ultra low-dose RFA induced a significant increase in CD4^+^ T-cell response relative to controls. Taken together, these data suggest that exposure to ultra low-dose RFA modulates tumors toward a more immunogenic phenotype and induces significant CEA-specific antitumor responses. Thus, it is feasible that clinical treatment of tumor lesions with RFA results in similar modulation at the thermal margin of the tumor [11].

### Different RFA Doses Distinctively Modulate Tumor Growth, Phenotype, and Immune Responses to Multiple TAAs

**Figure 4:**
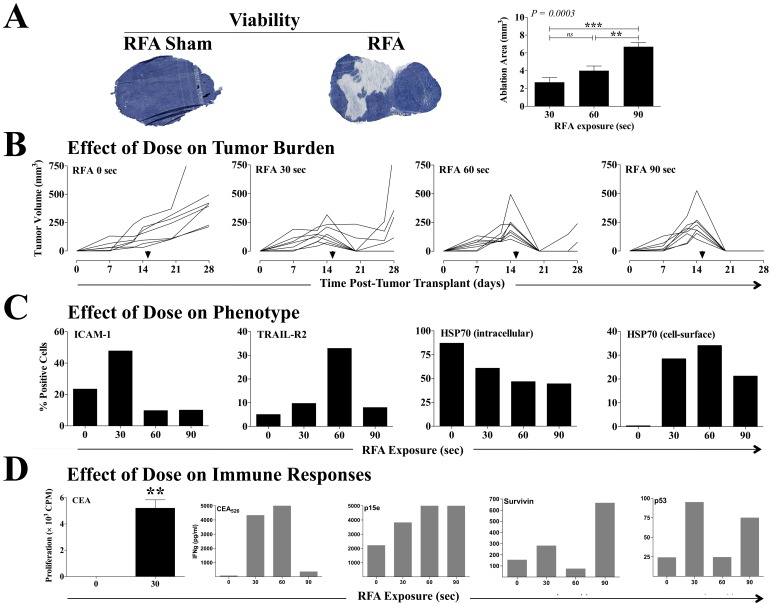
Effect of RFA dose on tumor phenotype and antigen cascade immune responses. Female C57BL/6 mice (*n* = 8–10/group) were injected s.c. on day 0 with MC38-CEA^+^ tumor cells. On day 15, tumors were exposed to RFA sham or to increasing doses of RFA (30, 60, or 90 s). **A**, 3 h post-RFA, tumor viability was assessed by tetrazolium staining (*n* = 4–7) in tumors exposed to sham or 30 s RFA. Histological images are representative of each treatment. Dark blue denotes viable cells. **B** depicts tumor volumes in individual animals. Arrows denote RFA on day 15. **C**, on day 17, excised CEA^+^ tumor cells were evaluated by flow cytometry for percentage of cells with cell-surface expression of ICAM-1, TRAIL-R2, and HSP70, and intracellular expression of HSP70. **D**, on day 29, purified CD4^+^ splenic T cells from animals exposed to RFA were tested for specific reactivity to CEA protein (25 ug/mL) in an *in vitro* lymphoproliferation assay. Results are depicted as mean CD4^+^ proliferation ± S.E.M. after subtraction of background CD4^+^ reactivity to control beta-galactosidase protein. Asterisks denote statistical significance (*P = *0.001, 2-tailed *t* test). CD8^+^ T-cell responses specific for the peptides CEA_526_, p15e_604_, p53_232_, and survivin_57_ were evaluated in pooled splenocytes through quantification of secreted IFN-g over 24 h of *in vitro* restimulation. Results are depicted as IFN-g (pg/mL) after subtraction of background IFN-g secretion in response to the control peptide VSV-N_52_. This experiment was performed twice yielding similar results.

To further investigate the spectrum of immunomodulatory events resulting from the RFA-induced gradient of thermal stress, we examined the effects on tumor of exposure to higher, clinically relevant doses of RFA. Female C57BL/6 mice transplanted with MC38-CEA^+^ s.c. tumors were exposed to sham RFA or to low (30 s), medium (60 s), or high (90 s) doses of RFA 15 days after tumor transplant. A cohort of animals (*n* = 4–7/group) was sacrificed 3 h post-RFA, and cellular viability in excised tumors exposed to sham or low dose RFA was assessed in histological sections by tetrazolium staining. As shown in Fig. 4A, exposure to RFA resulted in fewer tetrazolium^+^ tumor cells (dark blue), indicating a loss of viability. Quantification of tetrazolium^–^ cells (light blue) indicated that exposure to RFA induced significant loss of tumor-cell viability in a dose-dependent manner, suggesting that our RFA murine model could mimic clinical outcomes (Fig. 4A, right panel). To further examine the role of dose on treatment outcome, tumor volumes in individual animals were monitored for 28 days. As shown in Fig. 4B, RFA sham (0 s) had no effect on tumor growth and no complete response (CR) (defined as tumor eradication). Low-dose RFA (30 s) reduced tumor growth and resulted in a CR rate of 75%; however, 33% of eradicated tumors relapsed. Although 100% of tumors treated with a medium dose of RFA (60 s) attained a CR, 29% relapsed. The rate of CR also reached 100% with maximum RFA exposure (90 s), and there were no relapses. These data suggest that exposing MC38-CEA^+^ s.c. tumors to RFA mimics a wide spectrum of clinical outcomes, ranging from treatment failure to full treatment success, translated as CR.

Next, the immunomodulatory effects of distinct RFA doses were examined. Two days post-exposure to RFA sham or low-dose (30 s), medium-dose (60 s), or high-dose (90 s) RFA, excised CEA^+^ tumor cells were evaluated by flow cytometry for expression of ICAM-1, TRAIL-R2, MHC class I, Fas, and HSP70 proteins. Each of these molecules has been shown to enhance antitumor T-cell responses through diverse mechanisms [10], [34], [37], [38]. As shown in Fig. 4C, exposure to low-dose RFA doubled the population of cells with cell-surface expression of ICAM-1 relative to RFA sham control tumors. However, mean fluorescence intensity (MFI) of ICAM-1^+^ cells decreased 38% after exposure to low-dose RFA relative to controls (data not shown), suggesting an increase in ICAM-1 translation favoring cells with low endogenous ICAM-1 expression. Similar observations were attained with TRAIL-R2. The population of CEA^+^ cells expressing this receptor increased 2-fold with low-dose RFA compared to tumors receiving RFA sham. However, TRAIL-R2 MFI decreased 46% after exposure to low-dose RFA relative to controls (data not shown). After exposure to medium-dose RFA, the population of cells with cell-surface expression of TRAIL-R2 increased 7-fold with MFI decreasing 9% compared to controls. No changes in MHC class I or Fas expression was observed with the RFA doses used (data not shown).

Mounting evidence indicates that tumor cells exposed to thermal stress increase the expression of total HSP70 [39], [40]. This protein is constitutively expressed intracellularly in most cancer cells, where it functions as anti-apoptotic, and has been shown to bind to TRAIL receptors, thus inhibiting TRAIL-mediated cell death [37]. Intracellular HSP70 expression has also been associated with poor prognosis and resistance to both chemotherapy and radiation therapy [41]. In contrast, HSP70 expressed on the tumor cell surface has been shown to promote innate and adaptive immune responses, including enhanced T-cell activation and promotion of CD4^+^ and CD8^+^ cytotoxic immune responses [4], [38]. Thus, to further examine the effect of RFA dose on immune modulation of tumor, we examined intracellular and cell-surface expression of HSP70. As shown in Fig. 4C, exposure to RFA induced a dose-dependent decrease in intracellular HSP70 from >90% (RFA sham) to <50% (high-dose RFA) without affecting cellular MFI (data not shown). Further, RFA treatment translated into increase in HSP70 translocation to the tumor cell surface with all RFA doses relative to sham controls. Less than 2% of tumors treated with RFA sham exhibited HSP70 on their surface. This level increased to 34% with medium-dose RFA with a concomitant 349% increase in cellular MFI. Increases in cellular cell-surface HSP70 MFI ^3^370% were also observed in tumors treated with both low and high dose RFA (data not shown), suggesting that RFA modulates tumor phenotype into a more immunogenic platform.

In this context, we examined the effect of different RFA doses on the generation of CD4^+^ and CD8^+^ T-cell responses. Purified CD4^+^ splenic T cells were tested for reactivity *in vitro* to CEA protein 29 days post-tumor transplant. Compared to sham RFA, exposure to low-dose RFA (30 s) significantly increased proliferation of CD4^+^ T cells in response to CEA (*P* = 0.001) (Fig. 4D, left panel). CD8^+^ T-cell responses specific for the peptides CEA_526_, p15e_604_, survivin_57_, and p53_232_ were evaluated in pooled splenocytes by quantifying secreted IFN-γ over 24 h of restimulation *in vitro*. With varying dose-response profiles and magnitudes (Fig. 4D), RFA increased CD8^+^ IFN-γ secretion in response to CEA, p15e, survivin, and p53. These data suggest that RFA alone can induce a more immunogenic tumor phenotype, as well as a plethora of immune responses to multiple TAAs. Further, these data suggest that the immunogenic modulation profile of tumor cells at the thermal margin of a RFA ablated zone and its resulting contribution to the overall anti-tumor immune response will have distinct patterns based on the level of thermal stress received during the RFA treatment.

### Combination Therapy with RFA Plus Vaccine Alters Tumor Biology Toward a Less Invasive Signature through Modulation of MicroRNA Transcripts

The role of microRNAs in tumor response to energy-based therapies, including radiation, is under active investigation [42], [43]. MicroRNAs play a key role in regulating gene expression through post-transcriptional interference of messenger RNAs and can have various functions, including tumor suppression. Thus, to identify molecular signatures that could potentially be involved with RFA-induced antitumor immunity, a focused microRNA (miR) analysis of excised tumors was performed. On day 13 post-tumor transplant, CEA-Tg mice with established MC38-CEA^+^ tumors were exposed to RFA sham, high-dose RFA, vaccine alone, or vaccine combined with high-dose RFA. Tumors were harvested on day 16 and 88 microRNA transcripts were examined (Table S1). Transcripts were considered differentially expressed if their levels of expression differed ≥5-fold in any of the 3 treatment modalities compared with matched RFA sham controls. As shown in Table 2, 9 microRNAs were modulated by vaccine, RFA and/or RFA combined with vaccine based on this stringent criterion. Interestingly, all microRNAs differentially regulated by treatment have been shown to be tumor-suppressive [44], [45], [46], [47], [48], [49]. Vaccine alone increased the expression of miR-142-5p and miR-150, and downregulated miR-133b and miR-1. RFA alone induced up-regulation of 5 specific microRNA transcripts relative to treatment control: miR-1, -133b, -150, -203, and -205. However, in tumors exposed to RFA plus vaccine we observed a significant increase in microRNA expression of 7 of 9 transcripts relative to control tumors. Further, of the 5 microRNAs whose expression was modulated by both RFA alone and/or RFA combined with vaccine indicated that combination therapy induced a robust up-regulation of 3 of 5 tumor-suppressor transcripts. Strikingly, RFA alone increased miR-133b 13.3-fold, whereas combination therapy markedly decreased its expression (−30.1-fold) relative to control tumors. Similar results were observed with miR-1. Expression of miR-150 in MC38-CEA^+^ tumors increased 25.2-fold after exposure to RFA alone, and 32-fold after exposure to RFA plus vaccine. Expression of miR-363, -124, -142-5p, and -141 in tumors exposed to RFA alone did not differ from that of control tumors. However, all 4 microRNAs were markedly up-regulated after exposure to RFA plus vaccine. These data suggest that combination therapy with RFA plus vaccine modulates tumor biology toward a less invasive, less metastatic signature by synergistically increasing the expression of tumor-suppressive microRNAs.

**Table 2:** *In vivo* modulation of tumor suppressor microRNA transcripts in tumors exposed to vaccine, RFA alone, or RFA combined with vaccine.

Accession Number	microRNA	Vaccine	RFA	Vaccine+RFA
MIMAT0000134	mmu-miR-124	1	1	5.1
MIMAT0000154	mmu-miR-142-5p	6.2	1	5.3
MIMAT0000708	mmu-miR-363	1	1	6.1
MIMAT0000153	mmu-miR-141	1	1	13.6
MIMAT0000236	mmu-miR-203	1	5.4	13.7
MIMAT0000238	mmu-miR-205	1	6.0	44.8
MIMAT0000769	mmu-miR-133b	−10.0	13.3	−30.1
MIMAT0000160	mmu-miR-150	31.7	25.2	32.0
MIMAT0000123	mmu-miR-1	−19.6	73.2	−38.7

CEA-Tg mice (*n* = 3) received MC38-CEA^+^ cells on day 0. Tumors were exposed to RFA sham or intermediate-dose RFA (30 s; 60–70°C) on day 13. Vaccinated animals received rMVA-CEA/TRICOM on day 4, and rF-CEA/TRICOM on day 11, alone, or in combination with RFA. On day 16, tumors were harvested and microRNA array analysis was performed. Transcripts were considered differentially expressed (>1) if their levels of expression differed at least 5-fold in either treatment modality as compared with matched RFA sham control tumors. Accession numbers of microRNA transcripts refer to mirBase v14.0.

### RFA Plus Vaccine Enhanced CEA-Specific T-Cell Responses and Induced Abscopal Regression of Antigen-Disparate Tumors in a Metachronous Murine Model of Colon Carcinoma

**Figure 5:**
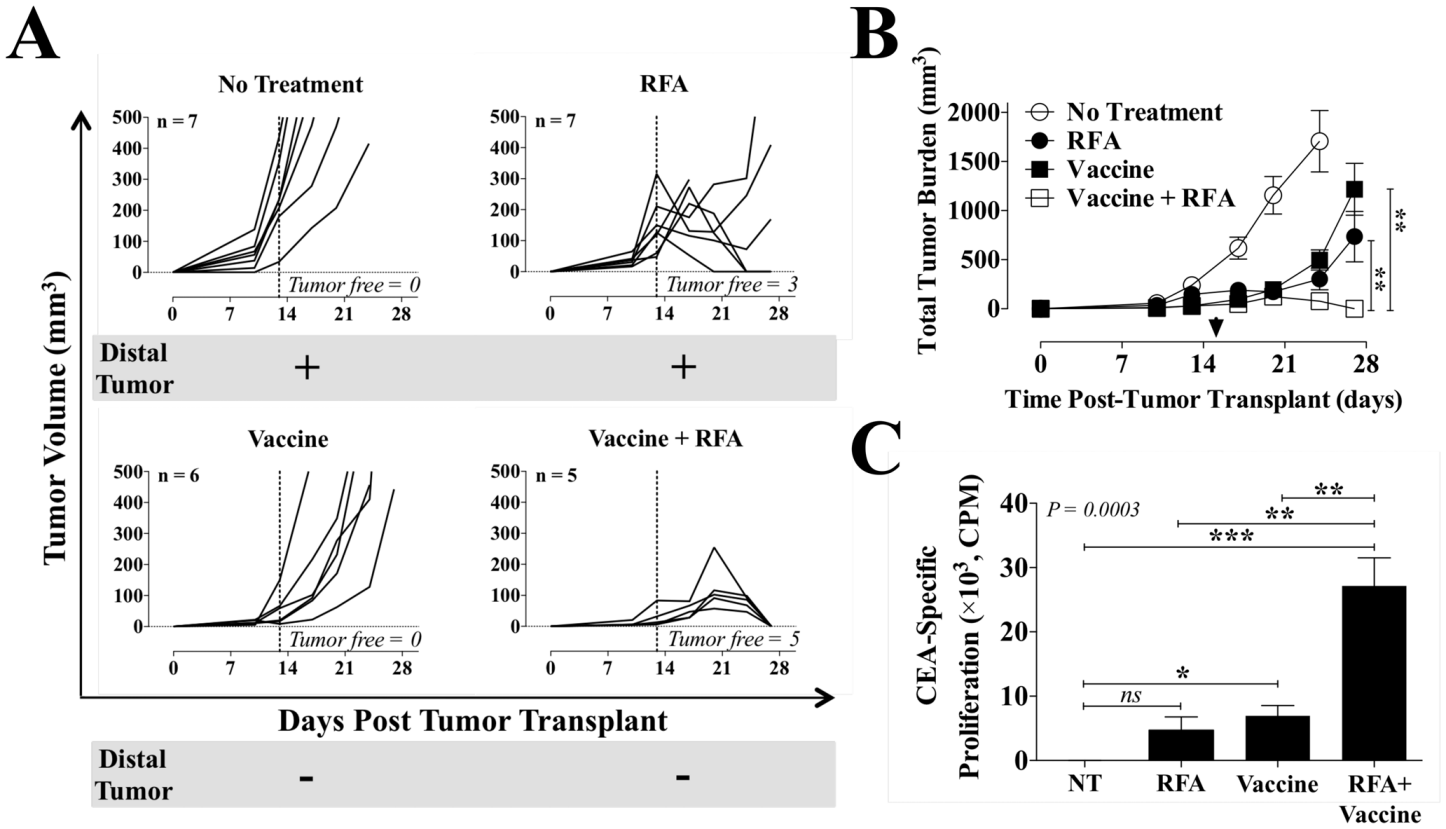
Antitumor efficacy and immune response elicited by combination therapy with RFA and recombinant vaccine. CEA-Tg mice (*n* = 5–7/group) received MC38-CEA^+^ cells on day 0 (right flank s.c.; primary tumor) and CEA^–^ MC38 cells on day 5 (left flank s.c.; distal tumor). A, primary tumor growth. MC38-CEA^+^ tumors received sham or high-dose RFA (30–300 s; 60–70°C) on day 13 (dotted line). Vaccinated animals received rV-CEA/TRICOM+rF-GM-CSF on day 4 and rF-CEA/TRICOM+rF-GM-CSF on days 11 and 18, alone or in combination with RFA. Each graph denotes the number of primary tumor-free mice and the presence (+) or absence (–) of a distal tumor on day 28. B, total tumor burden (primary plus distal). Asterisks denote statistical significance among treatment groups at day 27 (*P*<0.0001, 1-way ANOVA with Tukey’s multiple comparison test). Arrow indicates RFA treatment on day 15. C, CEA-specific proliferation of CD4^+^ T cells. CEA-Tg mice were transplanted with MC38-CEA^+^ tumor cells as before and received sham or 30 s RFA on day 15. Vaccinated mice received rMVA-CEA/TRICOM on day 4 and rF-CEA/TRICOM on days 11 and 18, alone or in combination with 30 s RFA. On day 39, purified CD4^+^ splenic T cells were tested for reactivity to CEA protein (50 ug/mL) in an *in vitro* lymphoproliferation assay. Results are depicted as mean CEA-specific CD4^+^ proliferation ± S.E.M. after subtraction of background CD4^+^ reactivity to control beta-galactosidase protein. Asterisks denote statistical significance among treatment groups (*P* = 0.0003, 1-way ANOVA with Tukey’s multiple comparison test). This experiment was performed twice yielding similar results.

Localized energy delivery such as tumor irradiation has been shown to induce a systemic antitumor immune response synergistic with vaccine, which translates into effective abscopal regression of distant metastases [50]. We investigated whether RFA-induced systemic immunity could synergize with a vaccine encoding CEA to promote antitumor efficacy and abscopal regression of CEA^–^ distal metastases in the setting of a self-antigen. We designed a metachronous tumor model in which mice were transplanted with 2 antigen-disparate tumors in discrete sites. CEA-Tg mice were transplanted with primary CEA^+^ tumors (MC38-CEA^+^ cells) on day 0, and distal CEA^–^ tumors (MC38 cells) on day 5. CEA^+^ tumors received sham or high-dose RFA on day 13. Vaccinated animals received vaccine weekly starting on day 4, alone or in sequential combination with high-dose RFA. As shown in Fig. 5A, RFA sham had no impact on regression of either primary or distal tumors. Treatment of the primary tumor with RFA alone resulted in a 43% CR rate, with 3 of 7 animals tumor-free after ablation. However, RFA alone was not able to eradicate the CEA^–^ distal tumor. No CR or regression of the primary tumor were observed in animals receiving vaccine alone. However, vaccine alone prevented the development of secondary tumors, which was also observed in animals receiving RFA plus vaccine, confirming previous observations in this tumor model (results not shown). Moreover, combination therapy eliminated 100% of primary CEA^+^ tumors, indicating that RFA synergizes with vaccine to promote effective antitumor immune responses, confirming previous observations from single-tumor models (not shown). Total tumor burden was calculated to better capture the overall antitumor effects of each treatment in both primary and distal tumors. Animals treated with vaccine, RFA, or vaccine plus RFA had a significant reduction in total tumor burden (*P*<0.0001) at day 24 (Fig. 5B). Further, treatment with RFA plus vaccine resulted in significant reduction of total tumor burden relative to vaccine or RFA alone (*P* = 0.0054).

To better understand the mechanisms involved in the antitumor efficacy mediated by vaccine plus RFA, CEA-Tg mice were transplanted with MC38-CEA^+^ tumor cells as before and received sham or RFA (30 s) on day 15. Vaccinated mice received rMVA-CEA/TRICOM on day 4 and rF-CEA/TRICOM on days 11 and 18, alone or in combination with RFA. On day 39, purified CD4^+^ splenic T cells from animals with similar tumor burden were tested for reactivity to CEA protein in an *in vitro* lymphoproliferation assay. As shown in Fig. 5C, control animals exposed to RFA sham had minimal CD4^+^-specific proliferation to CEA. RFA alone induced a higher CD4^+^ response, although not significantly different from that of controls. Vaccine alone was able to break immune tolerance to CEA and elicit a significant increase in CEA-specific CD4^+^ responses relative to RFA sham controls. However, CEA-specific CD4^+^ immune responses were highest in mice that received vaccine in combination with RFA vs. RFA alone, vaccine alone, or RFA sham (*P* = 0.0003). Taken together, these data indicate that RFA enhances CD4^+^ T-cell responses generated by CEA poxviral vaccines, which translates into a synergistic eradication of both CEA^+^ primary tumors and distal CEA^–^ metastases.

### Low- and High-Dose Sequential RFA Plus Vaccine Decreases Tumor Burden and Increases Relapse-Free Survival

**Figure 6:**
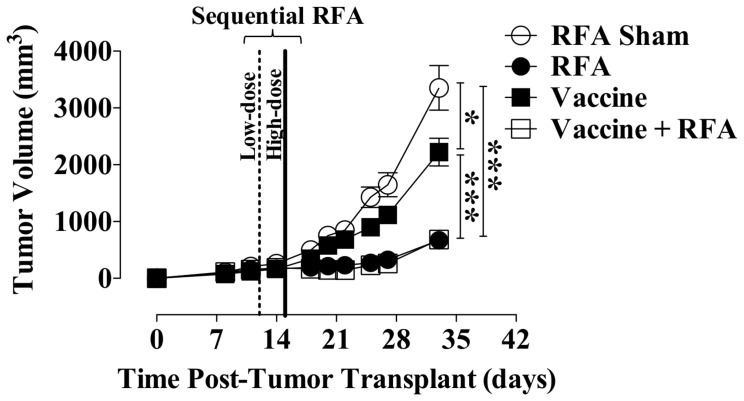
Antitumor efficacy of combination therapy with sequential RFA and recombinant vaccine. MC38-CEA^+^ cells were transplanted on day 0 on the right flank of CEA-Tg mice (*n* = 25–29/group). MC38-CEA^+^ tumors received sham or low-dose RFA (10–30 s at 70°C) on day 12 (dotted line), followed by high-dose ablative RFA (30–300 s at 70°C) on day 15 (solid line). Vaccinated mice received rMVA-CEA/TRICOM s.c. on day 4 and rF-CEA/TRICOM on day 11 and every 7 days thereafter, alone or in combination with sequential RFA. **A** depicts tumor growth. Data represent tumor volume ± S.E.M. in individual animals. Asterisks denote statistically significant differences among treatment groups (*P<*0.0001, 1-way ANOVA with Tukey’s multiple comparison test).

We hypothesized that combining vaccine with sequential RFA (low-dose immunogenic RFA followed by high-dose curative-intent RFA) would result in significant antitumor efficacy and improved relapse rates. To test this hypothesis, CEA-Tg mice received MC38-CEA^+^ cells on day 0. Tumors received sham or low-dose RFA on day 12, followed by ablative high-dose RFA on day 15 (defined as sequential RFA). Mice received vaccine weekly starting on day 4 in combination with sham or sequential RFA. Subsequent analysis of tumor volumes indicated significant differences among treatment groups (*P*<0.0001). Vaccine alone significantly decreased tumor burden relative to mice receiving RFA sham (Fig. 6) (*P*<0.05). Tumors exposed to sequential RFA alone or to combination therapy showed the highest decrease in tumor growth relative to controls (*P*<0.0001). Interestingly, sequential RFA plus vaccine did not decrease tumor volume more effectively than sequential RFA alone, confirming independent observations from two additional studies (data not shown). To better understand the antitumor effects of combination therapy relative to sequential RFA alone, we performed a focused analysis of CRs and durable CRs. In this context, any tumor eradication during the course of the study, regardless of relapse, was considered CR. Any tumor eradication without subsequent relapse during the course of the study was considered a durable CR. No CRs were observed in animals receiving RFA sham or vaccine alone (Table 3). During the course of this study, 34.5% of animals receiving sequential RFA attained a CR; 50% of these subsequently relapsed. However, animals receiving sequential RFA plus vaccine showed a higher CR rate, as well as increased relapse-free durable CR. Specifically, we observed that 52% of all animals receiving combination therapy became tumor-free. Further, 69.2% of all CRs in this cohort did not relapse. Thus, the combination therapy increased durable CRs by 19.2%. These results suggest a trend indicating that low-dose immunomodulatory RFA plus high-dose curative-intent RFA combined with vaccine may reduce the number of tumor relapses and thus prolong survival.

**Table 3:** Complete response rates to combination therapy with sequential RFA and recombinant vaccine.

Group	Complete Response	Durable Complete Response
RFA Sham	0	0
RFA	34.5% (10/29)	50% (5/10)
Vaccine+Sham	0	0
Vaccine+RFA	52% (13/25)	69.2% (9/13)
	*P = 0.153*	*P = 0.306*

MC38-CEA^+^ cells were transplanted on day 0 on the right flank of CEA-Tg mice (*n* = 25–29/group). MC38-CEA^+^ tumors received sham or low-dose RFA (10–30 s at 70°C) on day 12, followed by high-dose ablative RFA (30–300 s at 70°C) on day 15. Vaccinated mice received rMVA-CEA/TRICOM s.c. on day 4 and rF-CEA/TRICOM on day 11 and every 7 days thereafter, alone or in combination with sequential RFA. Results are depicted as percentage of mice with complete responses or durable complete responses following treatment with RFA sham, sequential RFA, vaccine combined with RFA sham, or vaccine with sequential RFA. Values in parentheses depict number of responders/number of eligible animals in each cohort. *P* values denote statistical analysis of response rates to sequential RFA alone or in combination with vaccine, as determined by contingency analysis (Fisher’s exact test).

## Discussion

*In situ* tumor ablation involves destroying tumor tissue with energy sources such as microwave, laser, cryotherapy, HIFU, or RFA. Each of these techniques relies on controlled energy delivery to minimize collateral damage to adjacent structures [2]. RFA is a minimally invasive technique and perhaps the most common choice for local tumor ablation as an alternative to surgical resection [1], [2]. RFA destroys tumor by delivering a radio-wave-frequency alternating current that generates frictional hyperthermia, causing cell death through coagulative necrosis [1], [4]. Mounting preclinical and clinical evidence indicates that RFA-induced necrosis is a source of tumor antigens, to which the host’s immune system can generate antitumor immunity superior to that elicited by surgical resection [5], [7], [51]. However, despite the increased availability of antigens, RFA of nonimmunogenic tumors does not usually induce systemic immunity [52]. Moreover, clinical reports indicate that RFA is not superior to surgical resection in the management of tumor recurrence, suggesting that the immune responses elicited by RFA alone are insufficient to prevent tumor recurrence. This has also been reported with other ablative modalities, including microwave, cryotherapy, and HIFU [1], [5], [8], [9].

Several therapeutic strategies have been employed to reduce local and systemic tumor relapse in patients treated with RFA, including combination with chemotherapy and various immunotherapies [53], [54], [55]. In a randomized trial (*n* = 95), patients with hepatocellular carcinoma (HCC) received chemoembolization sequentially combined with ablative RFA with or without subsequent adoptive immunotherapy in the form of autologous cytokine-induced killer cells. Tumor relapse was observed in 31% of patients receiving RFA plus immunotherapy compared to 85% of patients receiving RFA alone [54]. Similar rates of HCC recurrence were observed in patients receiving ablative RFA followed by IFN-alpha2b, where patients receiving combination therapy had a median tumor-free period of 3.4 years, a significantly higher progression-free survival than the 1.4 years for patients receiving RFA alone [55].

These encouraging results suggest that RFA combined with an immunotherapy regimen could prevent tumor relapse by augmenting and/or expanding RFA-induced immune responses. Thus, we hypothesized that combination therapy with RFA plus a vaccine targeting CEA, a widely expressed TAA, could have a synergistic antitumor effect and prevent tumor relapse. To test this hypothesis, we examined whether sublethal hyperthermia could induce a more productive immunogenic modulation, i.e., alter tumor phenotype to one more susceptible to immune attack. We have previously shown that tumors can be rendered more sensitive to immune-mediated lysis by sublethal exposure to selected chemotherapies and certain forms of energy such as gamma radiation [10], [24], [56]. Here we expand these observations to other treatment modalities based on thermal stress. We report that *in vitro* exposure of both murine and human colon carcinoma cells to sublethal thermal stress induces productive immunogenic modulation of tumor cells rendering them more sensitive to CTL-mediated killing, which was also observed with radiation (Figs. 1 and 2), a form of energy previously demonstrated to enhance tumor sensitivity to CTL-mediated lysis [24], [36]. We then investigated whether these findings could be replicated *in vivo* using ultra low doses of RFA. Our results (Fig. 3) confirmed that ultra low doses of RFA induce modulation of immune-relevant molecules within the tumor, resulting in a cascade of CEA-specific CD4^+^ T-cell immune responses. This confirms and extends observations that RFA and other forms of thermal ablation, such as HIFU, microwave, and cryotherapy, are able to induce tumor-specific immune responses [4], [57]. Further, when tumors were exposed to higher doses of RFA, mimicking the clinical spectrum of treatment success (Fig. 4), differential effects based on dose were observed in both modulation of tumor phenotype and generation of cascade T-cell responses to multiple TAAs. Strikingly, thermal stress and RFA dose modulated each immune-relevant protein differently. Although ICAM-1 expression was not modulated following hyperthermia in vitro (Table 1), it was significantly upregulated after exposure to both ultra low (7 s, Fig. 3) and low (30 s, Fig. 4) doses of RFA in vivo, which suggests a role for the tumor microenvironment in ICAM-1 regulation, in agreement with other reports [58]. Whereas tumor exposure to hyperthermia (Table 1) and to an ultra low dose of RFA (7 s, Fig. 3) markedly increased Fas and MHC class I expression, higher RFA doses (30–90 s, Fig. 4) had no effect on the cell-surface expression of these proteins. This indicates that the energy gradient delivered by RFA differentially modulates specific elements of tumor phenotype based on the dose of thermal stress delivered to each carcinoma cell, rendering the tumor more immune-sensitive in the absence of tumor eradication, and resulting in generation of CD4^+^ and CD8^+^ T-cell responses to a variety of TAAs, a finding supported by accumulating clinical evidence [4], [5], [6]. In clinical practice, it is feasible to deliver low dose RFA, followed sequentially by the more standard high dose and lethal RFA.

Mounting evidence indicates that tumor cells exposed to sublethal levels of heat or RFA undergo heat shock, leading to increased expression of total HSP70 at both the mRNA and protein levels [39], [40]. This protein is constitutively expressed intracellularly in most cancer cells, where it functions as an anti-apoptotic and has been shown to bind to TRAIL receptors, thus inhibiting TRAIL-mediated cell death [37]. Intracellular HSP70 expression has also been associated with poor prognosis and resistance to both chemotherapy and radiation therapy [41]. Importantly, we have observed that RFA reduces intracellular levels of HSP70 by promoting its translocation and that of TRAIL-R2 to the cell surface in a dose-response manner (Fig. 4C), which may make the tumor a better target for TRAIL-mediated immune attack. Moreover, membrane-bound or secreted HSP70 promotes increased innate and adaptive immune responses, cross-presents TAAs to antigen-presenting cells, enhances T-cell activation, and promotes CD4^+^ and CD8^+^ cytotoxic immune responses [59], [60]. We have also observed increased cell-surface expression of the T-cell adhesion molecule ICAM-1, as well as cascade CD4^+^ T-cell immune responses to CEA and CD8^+^ T-cell responses to CEA, p15e, survivin, and p53, indicating that RFA may induce a multitude of immune-relevant events in response to different doses that could potentially be exploited to promote effective antitumor immunity in combination with vaccine. These data are supported by clinical observations that development of strong CD8^+^ T-cell responses against TAAs prolongs recurrence-free survival after RFA in patients with HCC [61].

Analysis of microRNA transcripts (Table 2) supports and expands these observations. The role of microRNAs in tumor response to energy-based therapies, including radiation, is under active investigation [42], [43]. MicroRNAs play a key role in regulating gene expression through post-transcriptional interference of messenger RNAs and can have various functions, including tumor suppression. Here we report that tumor ablation with radiofrequency energy strongly increased the expression of several tumor-suppressor microRNAs, including miR-133b, which has been shown to inhibit cell growth in lung cancer [44]. Importantly, low expression of miR-133b in colorectal cancer has been correlated with metastases and lower rates of survival [62]. Although its significance is not yet clear, combination therapy with RFA and vaccine markedly decreased miR-133. Vaccine, RFA alone, and RFA combined with vaccine induced a robust increase in expression of miR-150, a tumor suppressor shown to inhibit cancer stem cells in pancreatic cancer [46]. Compared to RFA alone, the combination of RFA and vaccine further augmented and amplified the expression of shared and novel tumor-suppressor microRNA transcripts, including miR-141, a microRNA recently shown to inhibit migration and invasion in colorectal carcinoma cells [48], and miR-205, a tumor suppressor implicated in preventing epithelial-to-mesenchymal transition and whose repression has been associated with poor prognosis [45], [63]. The novel observation from these studies is that RFA plus vaccine significantly increases the expression of multiple tumor-suppressive microRNAs relative to RFA alone. This finding suggests that RFA and vaccine work synergistically to modulate tumor biology to make tumors more indolent and less metastatic. These results may provide a mechanistic rationale that supports clinical observations that combining RFA with immunotherapy delays tumor recurrence [55], [61]. However, further research is needed to confirm and determine whether this mechanism can be exploited and expanded to the combination of RFA and non-vaccine immunotherapy platforms.

Clinical reports have indicated that RFA can induce systemic immunity and control distant metastases following treatment of the primary tumor through a process known as the abscopal effect [26]. However, immune responses elicited by RFA alone are insufficient for both local and systemic control of tumors [8], [64], as indicated by frequent recurrence. Here we show (Fig. 5) that treatment with high-dose curative-intent RFA followed by administration of a vaccine targeting CEA results in antitumor efficacy. Although vaccine alone did not impact the growth of the primary tumor it had a marked abscopal effect on the development of the distal tumor, confirming previous observations in this tumor model (results not shown). This differential effect may be a consequence of study design as distal tumors were implanted 5 days after the primary tumor. In addition, vaccine prime was given at day 4, a time where the primary tumor is fully established. Our data further indicate that RFA enhances CD4^+^ T-cell responses generated by CEA viral vaccines (Fig. 5C). The result is a synergistic eradication of CEA^+^ primary tumors and a significant abscopal effect that mediates the elimination of distal CEA^–^ metastases through immune responses to non-vaccine encoded antigens, expanding similar findings observed with combination of vaccine and radiation [50].

We sought to further explore the antitumor synergy between RFA and vaccine by examining the immunogenic modulation of tumor induced by a regimen of low-dose RFA followed by high-dose ablative RFA plus a viral vaccine targeting CEA. Although the combination of sequential RFA with vaccine did not significantly decreased tumor burden relative to RFA alone (Fig. 6), the presence of vaccine increased the number of CRs and, more importantly, the number of durable CRs (Table 3). This suggests that while the combination of vaccine and this specific RFA regimen may not increase the rate of tumor eradication relative to RFA alone, it may work synergistically to prevent relapse. Several immune mechanisms may be associated with this outcome. Pretreatment with low-dose RFA not only makes the tumor a better target for CTL-mediated and, potentially, TRAIL-mediated immune attack (Figs. 3 and 4), but may also alter the tumor to become an *in situ* vaccine prime through HSP70 immune stimulation and initiation of significant CD4^+^ and CD8^+^ T-cell cascade immune responses to several TAAs not encoded by the vaccine. Low dose RFA when used in combination with vaccine should be further studied *in vivo*, or in the clinic, to assess whether the RFA algorithm could be modified to allow for a slower treatment, where high dose standard lethal RFA is preceded by low dose RFA. Tumor ablation with high-dose RFA has been shown to create an antigen sink for the generation of antitumor immunity [7]. Thus, sequential exposure to high-dose ablative RFA may act as a vaccine boost by increasing tumor necrosis, resulting in heightened release of antigens, including HSP70. Radiofrequency energy is currently delivered in a standard fashion, fairly rapidly, and without a slow ramping (like low dose RFA). RFA algorithms should be further studied to clarify what is the optimal fashion to ramp energy when RFA is used in combination with vaccine in order to maximize immune responses.

Our results show that RFA induces a plethora of immune events based on dose, including immunogenic modulation of tumor and CD4^+^ and CD8^+^ T-cell responses. Although the immune responses elicited by RFA alone may not be sufficient to prevent tumor recurrence, exploiting these immune events by combining RFA with vaccine has shown the potential to improve CR rates and prevent tumor recurrence. RFA can be applied to a wide range of malignancies that express CEA, including HCC, metastatic liver cancer, and renal cell, lung, and prostate carcinomas. We thus envision a wide array of potential clinical applications for the combined use of RFA with viral vaccines targeting CEA [65].

## Supporting Information

Figure S1 **Effect of hyperthermia on cellular growth of murine colon carcinoma cells.** MC38-CEA^+^ cells were exposed *in vitro* to 37°C or 42°C for 1 h. Tumor cells were incubated at 37°C/5%CO_2_ for 4 additional days. Cells were harvested daily and viable cells were counted by trypan blue exclusion. Results are presented as fold increase in cell number relative to day zero ± S.E.M. from 2 replicate flasks. Asterisks denote statistical significance (*P*<0.05) relative to control cells (2-tailed *t* test). This experiment was performed once.
(TIF)

Table S1 (DOC)
