# Supplementary material for: Combination Therapy with Local Radiofrequency Ablation and Systemic Vaccine Enhances Antitumor Immunity and Mediates Local and Distal Tumor Regression
Source: PLoS One. 2013 Jul 24;8(7):e70417. doi: 10.1371/journal.pone.0070417 (PMC3722166; doi:10.1371/journal.pone.0070417)
Supplement: Table S1 — (DOC) [file pone.0070417.s002.doc]

**TABLE S1. *In vivo* modulation of microRNA transcripts in tumors exposed to vaccine alone, RFA alone, or combination of RFA with vaccine.**

|  |  | **Fold change relative to RFA Sham** | | |
| --- | --- | --- | --- | --- |
| **mirBase v14.0 Accession #** | **miRNA ID** | **Vaccine** | **RFA** | **Vaccine + RFA** |
| MIMAT0000521 | mmu-let-7a | 1.74 | 1.53 | 1.14 |
| MIMAT0000769 | mmu-miR-133b | -9.99 | 13.27 | -30.10 |
| MIMAT0000246 | mmu-miR-122 | 2.39 | 1.90 | 2.38 |
| MIMAT0003187 | mmu-miR-20b | 1.68 | 2.04 | 2.09 |
| MIMAT0000766 | mmu-miR-335-5p | 1.06 | -1.10 | 1.11 |
| MIMAT0000518 | mmu-miR-196a | 1.61 | 1.38 | 1.76 |
| MIMAT0000135 | mmu-miR-125a-5p | 2.41 | 1.72 | 2.27 |
| MIMAT0000154 | mmu-miR-142-5p | 6.16 | 4.39 | 5.25 |
| MIMAT0000541 | mmu-miR-96 | 2.53 | 2.00 | 3.14 |
| MIMAT0000670 | mmu-miR-222 | 2.27 | 1.80 | 1.90 |
| MIMAT0000580 | mmu-miR-148b | 2.10 | 1.52 | 1.72 |
| MIMAT0000539 | mmu-miR-92a | 2.18 | 2.18 | 2.05 |
| MIMAT0000213 | mmu-miR-184 | 3.13 | 1.68 | 3.80 |
| MIMAT0000137 | mmu-miR-126-5p | 1.40 | 1.30 | 1.20 |
| MIMAT0000526 | mmu-miR-15a | 1.90 | 1.81 | 1.81 |
| MIMAT0000385 | mmu-miR-106a | 1.80 | 2.32 | 2.61 |
| MIMAT0003151 | mmu-miR-378 | 1.04 | 1.95 | -2.06 |
| MIMAT0000522 | mmu-let-7b | 2.28 | 1.42 | 1.33 |
| MIMAT0000238 | mmu-miR-205 | 3.02 | 6.04 | 44.77 |
| MIMAT0000149 | mmu-miR-137 | 1.72 | 1.80 | 2.88 |
| MIMAT0000141 | mmu-miR-130a | 2.52 | 2.06 | 2.04 |
| MIMAT0000229 | mmu-miR-199a-5p | 1.70 | 1.42 | 1.13 |
| MIMAT0000151 | mmu-miR-140 | 2.19 | 2.15 | 2.37 |
| MIMAT0000529 | mmu-miR-20a | 1.78 | 2.14 | 2.52 |
| MIMAT0003475 | mmu-miR-146b | 1.60 | 1.56 | 1.33 |
| MIMAT0000144 | mmu-miR-132 | 1.96 | 1.66 | 1.78 |
| MIMAT0004859 | mmu-miR-193b | 2.12 | 1.80 | 1.95 |
| MIMAT0000212 | mmu-miR-183 | 2.12 | 1.81 | 2.48 |
| MIMAT0000381 | mmu-miR-34c | 2.01 | 1.93 | 2.99 |
| MIMAT0000514 | mmu-miR-30c | 2.43 | 2.18 | 1.57 |
| MIMAT0000516 | mmu-miR-148a | 2.28 | 1.51 | 1.57 |
| MIMAT0000146 | mmu-miR-134 | 1.99 | -1.05 | 1.03 |
| MIMAT0000121 | mmu-let-7g | 1.84 | 1.95 | 1.78 |
| MIMAT0000150 | mmu-miR-138 | 2.18 | 1.85 | 2.33 |
| MIMAT0000153 | mmu-miR-141 | 3.33 | 2.20 | 13.57 |
| MIMAT0000523 | mmu-let-7c | 2.07 | 1.52 | 1.28 |
| MIMAT0000524 | mmu-let-7e | 1.87 | 1.51 | 1.24 |
| MIMAT0000663 | mmu-miR-218 | 1.34 | 1.59 | 1.62 |
| MIMAT0000127 | mmu-miR-29b | 2.01 | 2.15 | 2.43 |
| MIMAT0000158 | mmu-miR-146a | 3.57 | 3.61 | 2.62 |
| MIMAT0000659 | mmu-miR-212 | 1.91 | 2.34 | 2.94 |
| MIMAT0000612 | mmu-miR-135b | 1.86 | 2.06 | 1.61 |
| MIMAT0000538 | mmu-miR-31 | 1.99 | 2.07 | 2.51 |
| MIMAT0000134 | mmu-miR-124 | 1.91 | 1.82 | 5.05 |
| MIMAT0000530 | mmu-miR-21 | 1.81 | 2.01 | 2.17 |
| MIMAT0004324 | mmu-miR-181d | 1.78 | 1.63 | 1.81 |
| MIMAT0000379 | mmu-miR-301a | 1.77 | 1.86 | 2.64 |
| MIMAT0000225 | mmu-miR-195 | 2.19 | 2.15 | 1.86 |
| MIMAT0000655 | mmu-miR-100 | 1.84 | 1.64 | 1.47 |
| MIMAT0000208 | mmu-miR-10b | 1.60 | 1.45 | 1.40 |
| MIMAT0000165 | mmu-miR-155 | 2.62 | 2.42 | 2.45 |
| MIMAT0000123 | mmu-miR-1 | -19.61 | 73.23 | -38.70 |
| MIMAT0000708 | mmu-miR-363 | 4.42 | 4.49 | 6.13 |
| MIMAT0000160 | mmu-miR-150 | 31.66 | 25.20 | 31.95 |
| MIMAT0000122 | mmu-let-7i | 1.79 | 1.81 | 1.64 |
| MIMAT0000126 | mmu-miR-27b | 1.52 | 1.97 | 1.22 |
| MIMAT0000677 | mmu-miR-7a | 1.48 | 1.87 | 2.01 |
| MIMAT0000669 | mmu-miR-221 | 1.70 | 1.48 | 1.47 |
| MIMAT0000535 | mmu-miR-29a | 2.26 | 2.07 | 1.80 |
| MIMAT0000221 | mmu-miR-191 | 3.02 | 2.53 | 2.77 |
| MIMAT0000383 | mmu-let-7d | 1.99 | 1.88 | 1.36 |
| MIMAT0000142 | mmu-miR-9 | 1.45 | 1.80 | 2.65 |
| MIMAT0000525 | mmu-let-7f | 1.58 | 1.68 | 1.55 |
| MIMAT0000648 | mmu-miR-10a | 1.96 | 1.83 | 1.99 |
| MIMAT0000673 | mmu-miR-181b | 2.22 | 1.79 | 1.95 |
| MIMAT0000124 | mmu-miR-15b | 2.70 | 2.32 | 2.44 |
| MIMAT0000527 | mmu-miR-16 | 2.25 | 2.23 | 2.29 |
| MIMAT0000658 | mmu-miR-210 | 2.71 | 2.49 | 4.38 |
| MIMAT0000649 | mmu-miR-17 | 1.80 | 1.96 | 2.32 |
| MIMAT0000545 | mmu-miR-98 | 1.42 | 1.65 | 1.53 |
| MIMAT0000542 | mmu-miR-34a | 1.99 | 1.91 | 2.06 |
| MIMAT0000652 | mmu-miR-25 | 2.16 | 2.32 | 2.15 |
| MIMAT0000156 | mmu-miR-144 | 1.57 | 1.00 | 1.15 |
| MIMAT0000140 | mmu-miR-128 | 1.67 | 2.38 | 1.09 |
| MIMAT0000665 | mmu-miR-223 | 2.26 | 1.63 | 1.63 |
| MIMAT0000904 | mmu-miR-215 | 1.86 | 2.57 | 3.06 |
| MIMAT0000651 | mmu-miR-19a | 1.68 | 2.29 | 2.44 |
| MIMAT0004544 | mmu-miR-193* | 2.31 | 1.68 | 1.74 |
| MIMAT0000528 | mmu-miR-18a | 1.78 | 2.22 | 2.61 |
| MIMAT0000661 | mmu-miR-214 | 1.89 | 1.40 | 1.54 |
| MIMAT0000138 | mmu-miR-126-3p | 1.53 | 1.31 | 1.27 |
| MIMAT0000537 | mmu-miR-27a | 1.83 | 2.09 | 1.96 |
| MIMAT0000546 | mmu-miR-103 | 2.22 | 1.62 | 2.06 |
| MIMAT0000159 | mmu-miR-149 | 1.38 | 1.92 | 1.35 |
| MIMAT0000125 | mmu-miR-23b | 1.77 | 1.83 | 1.28 |
| MIMAT0000236 | mmu-miR-203 | 1.67 | 5.44 | 13.66 |
| MIMAT0000654 | mmu-miR-32 | 2.84 | 2.71 | 3.89 |
| MIMAT0000674 | mmu-miR-181c | 2.46 | 1.99 | 2.27 |

CEA-Tg mice (*n* = 3) received MC38-CEA^+^ cells on day 0. Tumors were exposed to RFA sham or intermediate-dose RFA (30 s; 60-70°C) on day 13. Vaccinated animals received rMVA-CEA/TRICOM on day 4, and rF-CEA/TRICOM on day 11, alone, or in combination with RFA. On day 16, tumors were harvested and microRNA array analysis was performed. Results are presented as fold change of each transcript in each treatment modality relative to matched RFA sham control tumors. Accession numbers of microRNA transcripts refer to mirBase v14.0.
